# Historical baselines in marine bioinvasions: Implications for policy and management

**DOI:** 10.1371/journal.pone.0202383

**Published:** 2018-08-16

**Authors:** Henn Ojaveer, Bella S. Galil, James T. Carlton, Heidi Alleway, Philippe Goulletquer, Maiju Lehtiniemi, Agnese Marchini, Whitman Miller, Anna Occhipinti-Ambrogi, Melita Peharda, Gregory M. Ruiz, Susan L. Williams, Anastasija Zaiko

**Affiliations:** 1 Estonian Marine Institute, University of Tartu, Pärnu, Estonia; 2 The Steinhardt Museum of Natural History, Tel Aviv University, Tel Aviv, Israel; 3 Maritime Studies Program of Williams College and Mystic Seaport, Mystic, Connecticut, United States of America; 4 School of Biological Sciences, University of Adelaide, Adelaide, Australia; 5 Scientific Direction, IFREMER, Nantes, France; 6 Marine Research Centre, Finnish Environment Institute, Helsinki, Finland; 7 Department of Earth and Environmental Sciences, University of Pavia, Pavia, Italy; 8 Marine Invasion Research Laboratory, Smithsonian Environmental Research Center, Edgewater, Maryland, United States of America; 9 Institute of Oceanography and Fisheries, Split, Croatia; 10 Bodega Marine Laboratory and Department of Evolution and Ecology, University of California at Davis, Bodega Bay, California, United States of America; 11 Coastal and Freshwater Group, Cawthron Institute, Nelson, New Zealand; 12 Marine Research Institute, Klaipeda University, Klaipeda, Lithuania; Universita degli Studi di Genova, ITALY

## Abstract

The human-mediated introduction of marine non-indigenous species is a centuries- if not millennia-old phenomenon, but was only recently acknowledged as a potent driver of change in the sea. We provide a synopsis of key historical milestones for marine bioinvasions, including timelines of (a) discovery and understanding of the invasion process, focusing on transfer mechanisms and outcomes, (b) methodologies used for detection and monitoring, (c) approaches to ecological impacts research, and (d) management and policy responses. Early (until the mid-1900s) marine bioinvasions were given little attention, and in a number of cases actively and routinely facilitated. Beginning in the second half of the 20^th^ century, several conspicuous non-indigenous species outbreaks with strong environmental, economic, and public health impacts raised widespread concerns and initiated shifts in public and scientific perceptions. These high-profile invasions led to policy documents and strategies to reduce the introduction and spread of non-indigenous species, although with significant time lags and limited success and focused on only a subset of transfer mechanisms. Integrated, multi-vector management within an ecosystem-based marine management context is urgently needed to address the complex interactions of natural and human pressures that drive invasions in marine ecosystems.

## Introduction

Marine ecosystems are affected by several well-known human-induced global pressures, such as exploitation of living resources, land-based pollution, eutrophication, physical destruction and climate change (e.g., [1,2]). Many studies have documented human-mediated introductions of non-indigenous species (NIS), yet only relatively recently NIS have been recognized as a major threat that may cause significant changes in the structure and function of marine ecosystems [3].

Multiple human-induced pressures, which vary across Earth’s oceans, interact in complex and often non-linear ways [4]. Evaluation of the cumulative effects is essential to successful ecosystem-based management (e.g., [5,6]). Although our ability to comprehend interactions between human pressures and evaluate their cumulative effects is improving, managerial response still mostly relies on sectoral approaches. Whereas alleviation of specific pressures (e.g., pollution, fisheries) have resulted in some instances in the improvement of local marine environments and their living resources [7], there is no such evidence available for bioinvasion management, where many historically well-documented regions with sound biodiversity baselines exhibit clear temporal increases in detection rates of new NIS introductions (e.g., [8,9]). We consider that ‘NIS remain NIS,’ regardless of the time passed since their first detected presence.

Herein we address the “shifting baseline” syndrome in marine bioinvasions. This syndrome was first recognized in fisheries science wherein the state of the fishery was assessed based on a contemporary stock size and species composition, overlooking the prior history of the fishery, leading to underestimation of the magnitude of change and the degree of overexploitation [10,11]. The extent of marine bioinvasions may be similarly occluded. Carlton [12] presented an overview of the taxonomic, historical, and shifting baseline impediments to understanding of marine bioinvasions. Over the past 30 years, invaluable historical overviews on marine bioinvasions have confirmed their ancient origins (e.g., [13–16]). The advancement and application of new molecular and genomic methods will continue broadening our view of past invasions (e.g., [17–19]). However, a lack of quantitative, high -resolution analyses and detection methods aimed at marine bioinvaders and their histories further deepens the “shifting baseline” syndrome effect, and prevents a more complete understanding and acknowledgment of the full extent of the problem.

This paper provides a synopsis of the essential aspects related to the history of marine bioinvasions globally, through collating and synthesizing information on i) early evidence of species introductions by different vectors, ii) dynamics of introduction vectors and human perceptions over time, and iii) evolution of methodologies used for detection, identification and surveillance. We frame the assembled historical information into policy and management perspectives through i) outlining milestones in relevant policy and management acts and ii) making broad comparisons among the vector dynamics in the recent past and the content and efficacy of legislative management acts. In doing so we identify key messages crucial to the effective management of NIS, as well as redress some of the historical legacies.

## A history of vectors dynamics and associated introductions

### Vessels

#### Early shipping

Throughout history, the maritime shipping has played a fundamental role as means of transportation of goods and people [20–22]. However, we know little of the relationship between the early sea voyages and the dispersal of species *on* (as fouling communities), *in* (as boring communities) and *inside* (as ballast communities) ancient wooden sailing ships. We do know that there were extensive biofouling communities on these vessels, that shipworms were known to the ancients, and that solid ballast was loaded into ships since the Bronze age. It is highly likely that the dispersal and introduction of marine animals and plants by sea-going ships, in hull fouling and in damp rock-, shingle-, and sand- ballasted holds, commenced long ago, millennia before marine biologists began documenting the biogeography of organisms [12]. Persuasive insights and a strong signal into the probable scale of early invasions comes from the archaeo-entomologists who have traced the expansion of the European insect fauna *via* Roman and Viking ships around Europe and across the Atlantic Ocean ([23,24] and references therein). The same ships transporting terrestrial life would, of course, have transported marine life as well. A compelling example of an ancient invasion is the North American clam *Mya arenaria*. No fossil record is known in Europe, where it likely appeared by the 1200s ([25,26], see also Table 1).

**Table 1:** Examples of evidence of early introductions of selected marine non-indigenous species.

Taxon	Species	First detected presence	Origin to recipient region	Likely vector	Reference
Mollusca: Bivalvia	*Mya arenaria* (soft-shelled clam)	1200s	North America to Europe	Hull fouling, rock ballast	[25,27]
	*Mytilus* spp. (mussels)	1500s	Northern hemisphere to South America	Hull fouling	[14]
	*Crassostrea angulata* (oyster)	1500s	Western North Pacific to Southern Europe	Hull fouling, ballast	[12]
Mollusca: Gastropoda	*Littorina saxatilis* (rock periwinkle)	1792	Western Europe to Adriatic Sea	Rock ballast	[28]
	*Littorina littorea* (shore periwinkle)	1840s	Europe to North America	Rock ballast	[29]
Crustacea: Brachyura	*Carcinus maenas* (green crab)	1817	Europe to North America	Hull fouling, rock ballast	[30,31]
Crustacea: Isopoda	*Sphaeroma terebrans* (pill bug)	1860s	Indian Ocean to Brazil	Ship hull fouling or boring	[32]
Plantae: Chlorophyta	*Halimeda opuntia* (green alga)	1699	Indo-West Pacific to the Caribbean	Hull fouling	[33]
Plantae: Tracheophyta	*Spartina alterniflora* (cordgrass)	1803	North America to France	Shore ballast	[34]

Shipping expanded dramatically in the late 1500s [26]. However, as with antiquity, we have limited insight into marine bioinvasions of this era. Both vessel hull fouling and ballast likely played significant roles. Lindroth [35] notes that solid ballast discharge regulations were already in place by 1611 in the New World, which suggests the early awareness of the sheer volume of ballast being transported. Carlton and Hodder [36] undertook the first experimental studies on the fouling communities on a vessel in transit, focusing on a replica of a 16th century sailing ship, and thus providing insights into what may have been transported by vessels in the 1500s. The vessel sequentially accumulated species along the voyage route, such that it arrived in one port with species accumulated from previous ports (harbors) of call. This vessel also sank into mud at low tide in one port, acquiring benthic species not normally thought to have been transported by ships. In addition, Carlton [37] reconstructed the potential assemblage of marine animals and plants that may have been transported by a wooden sailing ship of 1750, suggesting that two dozen or more species (certainly an underestimate) could have been transported in ballast alone. However, we have no early records of the fauna transported by ballast, and only limited records of the flora, thanks to 19^th^ century sampling of the latter, known as “ballast waifs”, on ballast dumping grounds [38].

Records of ballast-mediated introductions begin to appear by the late 1700s and early 1800s. The type specimen of one of the world's best-known salt marsh plants, the North American *Spartina alterniflora*, was collected in France in 1803 [34], and thus likely introduced to the region in the 1700s in ships' ballast. It was transported to South America by 1817 either from North America or Europe. As an ecosystem engineer, it caused profound changes on the west coast of South America: marshes now occupy vast areas where mudflats used to exist, with concomitant changes in bird, fish, and invertebrate diversity and trophic relationships [34].

Rock ballast was the probable vector for the arrival and spread of the European periwinkle *Littorina littorea* in North America. This well-known snail is one of the most meticulously documented invasions of the early 1800s [29,39,40]. The large-shelled, intertidal marine molluscan fauna of Eastern North America (present day Canada and the United States) was already reasonably well known to European scientists by the mid- to late-1700s, such that the discovery of this western European snail *L*. *littorea* in Nova Scotia *circa* 1840s was greeted with a good deal of surprise by British scientists. Its southward spread over the following decades to the mid-Atlantic coast has been well documented. Through detailed investigation of shipping and ballast history commencing in the 1770s, Brawley et al. [40] linked the introduction of both *L*. *littorea* and the European seaweed *Fucus serratus* (in the 1860s) to the discharge of solid ballast from Western Europe. Carlton [41] noted that the invasion of this small snail effectively re-organized the structure and function of rocky, soft bottom, and salt marsh intertidal shores of the Northwest Atlantic Ocean. Even before *L*. *littorea* appeared in North America, *Littorina saxatilis* was carried by rock ballast to the Adriatic Sea, where it was found to be established by 1792 [28]. The same era saw the ship-mediated arrival in North America of the European green crab *Carcinus maenas* [42], which became one of the major shoreline predators of the Atlantic seaboard.

#### Modern shipping

**Fig 1:**
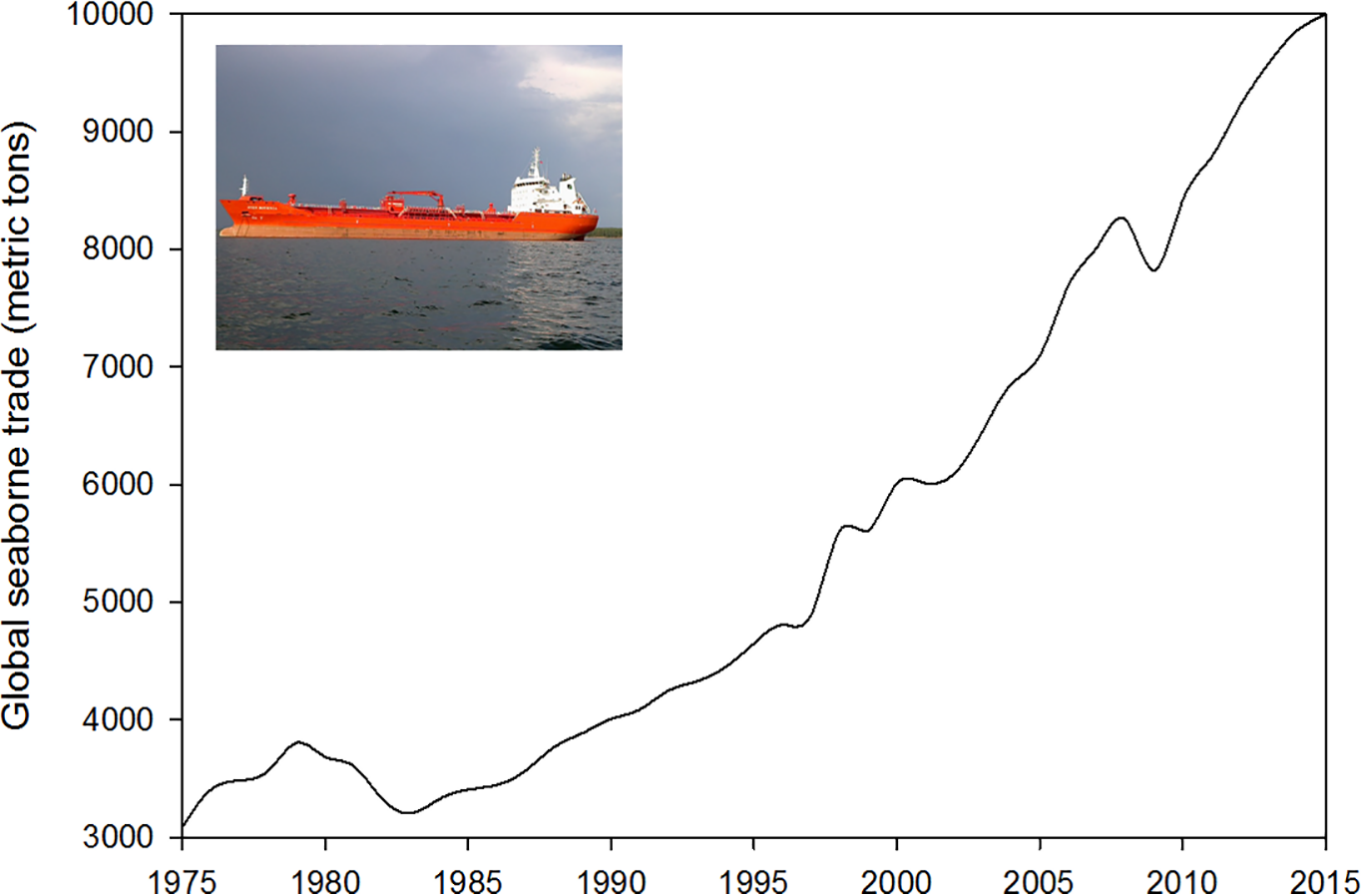
Global seaborne trade, volume in metric tons, 1975–2015 (data from [45]). Photo credit: Maiju Lehtiniemi.

The 19^th^ and 20^th^ centuries saw key innovations to ship design and manufacturing (e.g., engine powered steel-hulled vessels) which resulted in major changes in ship operations and behavior [43]. As markets became increasingly globalized, shipping volumes soared. The massive increase in shipping since the 1950s, boosted by the development of container-shipping in the 1960s [44], underpins the growth in world trade. According to data from the United Nations Conference on Trade and Development [45], global seaborne trade has increased by 3.8 times from 1970 to 2015, exceeding 9 billion tonnes loaded worldwide in 2015 (Fig 1) with developing countries increasingly contributing to the total volumes of international seaborne trade [45].

Global shipping routes have evolved since the end of the 20th century, shifting from one based on direct port to port services along the major East–West routes, which linked the three poles of the global economy (Europe, the United States and East Asia), to a ‘hub and spoke’ network, linking the major East–West maritime motorway with the secondary North–South services [46]. This shift in trade routes has functionally increased direct and indirect connectivity among global ports and harbors [47], whereby a decade ago approximately ten billion tonnes of ballast water were transported around the world by ships annually [48]. As vessel size, speed and number increased, so too have the likely number of organisms transported alive across oceans in hull fouling and ballast. For example, an early study of ships’ ballast water entering the North American Great Lakes revealed an average of 17 live species with densities varying from 10,000 to 8 billion individuals per vessel [49]. Additional studies have further demonstrated the magnitude and diversity of marine organisms delivered in ballast throughout the world [50]. In addition to ballast, slow speed transits of recreational vessels, drilling rigs, barges and floating docks have been documented to further contribute to the dispersal of a wide diversity of fouling organisms [51–54].

Despite the realization of such broad scale species transfers, it took a confluence of economically disastrous events to gain management response (see Global policy and legislation). Some of the high-impact examples include dinoflagellates, comb jellies and mussels. The ballast-water introduction of the carnivorous comb jelly *Mnemiopsis leidyi* into the Black Sea in the 1980s was associated with major ecosystem and severe adverse economic effects [55]. In the 1980s vessels entering the North American Great Lakes dumped ballast water from freshwater ports in Europe with propagules of the now notorious zebra mussel *Dreissena polymorpha* and quagga mussel *Dreissena bugensis*–quite likely the most economically and biologically disruptive NIS in North America [56]. Evidence from historic plankton samples, cyst surveys in sediment cores and genetic studies implicated ballast water as the source of introduction of the photosynthetic dinoflagellate *Gymnodinium catenatum* and the likely source of neurotoxic poisoning, leading to the closure of 15 shellfish farms for periods up to six months in Tasmania in the 1980s [57].

#### Recreational boating

The use of recreational craft is increasingly considered a high-risk vector for primary introductions and secondary spread of marine NIS, owing to their numbers, spatial distribution, travel patterns, and connectivity between high risk NIS hubs [58–62]. Mass marine recreational boating is a relatively recent phenomenon, initiated in the 1920s-30s and greatly expanded since the 1960s [63]. The number of coastal marinas grew from 5 in 1960 to 54 in 2000 in Queensland, Australia, and from 403 in 1985 to 716 in 2002 in Italy. In Florida and California, USA, in 2010, 914,535 and 810,008 boats, respectively, were registered [64]. In Ireland 29 marinas operated in the early 2000s, whereas none existed in mid-1970s [65]. Based on satellite images from 2007, the number of recreational boats in the Mediterranean Sea was approximately 1.5 million at the time [66].

Recreational craft are often moored for long periods and may accumulate organisms from the local fouling communities, transporting them to the next marina or mooring place, or to even distant ports. Largely overlooked, water entrained in bilge spaces during the transit also may contribute to spread of marine organisms [67]. In regions favoured by boaters (Caribbean Sea, Mediterranean Sea, and generally subtropical and temperate seas near affluent population centres), leisure craft provides high connectivity between high and low NIS locales (‘hub and spoke’), enhancing invasion risk by increasing potential propagule pressure [68]. But even in cold-temperate areas risks are high: in British Columbia, Canada, over a quarter of boats surveyed (25.7%) were fouled with one or more NIS [60,69].

Despite the growing number and geographical distribution of marinas and seaworthy leisure craft, investigations of introduction and translocation of NIS mediated by recreational boating only began in the 1990s (e.g., [70] and references therein), and to date the data remain geographically restricted, thus often underestimating the problem [71].

### Trade in live organisms

#### Culture

Farming of marine and partly marine (anadromous, catadromous) organisms (including fish, invertebrates and plants) for food and other products has a long history. Some target species are bred and raised in enclosed systems, whereas others are cultured to a certain life stage and placed in the sea in enclosures (cages, rafts), or released to roam freely. Farming is increasing to address the demand for marine food and to replace or restore declining coastal fisheries [72,73].

In the first century AD the Romans constructed *Ostriaria* for rearing of oysters [74], and transported them regionally within the Mediterranean Sea (e.g. from Brindisi in the southern Adriatic Sea to be reared in the Gulf of Baia in the Tyrrhenian Sea), in effect an early form of sea ranching [75]. Stock enhancement has been long practiced too: in the 11th century, “…*King Knud the Great brought oysters home from England and introduced them to the Wadden Sea*” [76].

The intentional transplantation of alien edible marine species in the late 19th century occurred partly in response to increased demand for seafood and to native stock failures. In 1860, the east Asian oyster *Crassostrea angulata* was imported to France from Portugal to compensate for shortage of seed of the native oyster *Ostrea edulis* [77], as well as the northern quahog *Mercenaria mercenaria* [78]. A century later, mass mortality of *C*. *angulata* triggered introduction of the Japanese cupped oyster *Crassostrea gigas* to France (Table 2; [79]). Of the current global production of *C*. *gigas*, about 15% originate from Europe and 7% from America [80]. Vast numbers of the North American Atlantic oyster *Crassostrea virginica* were transplanted in the 19th century to the American Pacific coast (see ‘Live seafood trade’), as well as released into European waters before marketing [81,82].

**Table 2:** Examples of records of four widely introduced non-indigenous cultured marine species.

First record	Country/region of origin	Country/region of introduction	Reference
*Crassostrea gigas* (Japanese cupped oyster)			
1902	Japan	USA: Washington State	[83]
1919	Japan	USA: Washington State	[84]
1925	Japan	Canada: British Columbia	[85]
1947	Japan	Australia: Tasmania	[86]
1966	Japan	France	[79]
1972	USA	French Polynesia	[87]
1973	France	South Africa: Cape Province	[88]
1975	Taiwan	USA: Guam	[89]
1982	Chile	Argentina	[90]
*Oncorhynchus mykiss* (Rainbow trout)			
1882	USA: Pacific coast	Germany	[91]
1884	USA: Atlantic coast	Belgium	[91]
1890	Russia	Lithuania	[91]
1898	Germany	Finland	[91]
1902	Northeast Pacific	Norway	[91]
1983	Northeast Pacific	Iceland	[91]
*Penaeus vannamei* (Whiteleg shrimp)			
1972	Mexico, Panama	New Caledonia	[92]
1978–1985	USA	USA: Hawaii	[93]
1985	Panama: Pacific coast	USA: South Carolina	[92]
1985	Panama: Pacific coast	Cuba	[94]
1988	USA: Texas, Hawaii	China	[92,95]
1997	Taiwan	Philippines	[96]
1998	Taiwan	Thailand	[96]
2000	China	Vietnam	[96]
2001	Taiwan	India	[96]
*Ruditapes philippinarum* (Manila clam)			
1930s	Japan	USA/Canada: Pacific coast	[97]
1972	USA: Pacific coast	France	[98]
1980	USA: Pacific coast	England	[99]
1983	England	Italy: Adriatic Sea	[100]
1984	Spain	Portugal	[101]

Attempts to augment marine finfish production by releasing hatched larvae started in the 1870s, mainly using cod and plaice [102]. In the beginning of the 1880s marine hatcheries were built in Europe and North America, mainly rearing anadromous fish [103]. In the 20^th^ century, the Soviet Union pursued extensive marine fisheries enhancement (MFE) programs, introducing the king crab *Paralithodes camtschaticus* and pink salmon *Oncorhynchus gorbuscha* to the Barents Sea, and sturgeons (*Acipenser gueldenstaedtii*, *Huso huso*) and salmonids (*Coregonus baerii*, *O*. *gorbuscha*, *O*. *keta*) to the Baltic Sea, together with several mysids introduced to increase the diversity of fish diet [104–107]. Farming of non-indigenous salmonids continues to be widespread phenomenon—a sizable share of the global production of Atlantic salmon is now located in Chile and Tasmania, Australia [80].

**Fig 2:**
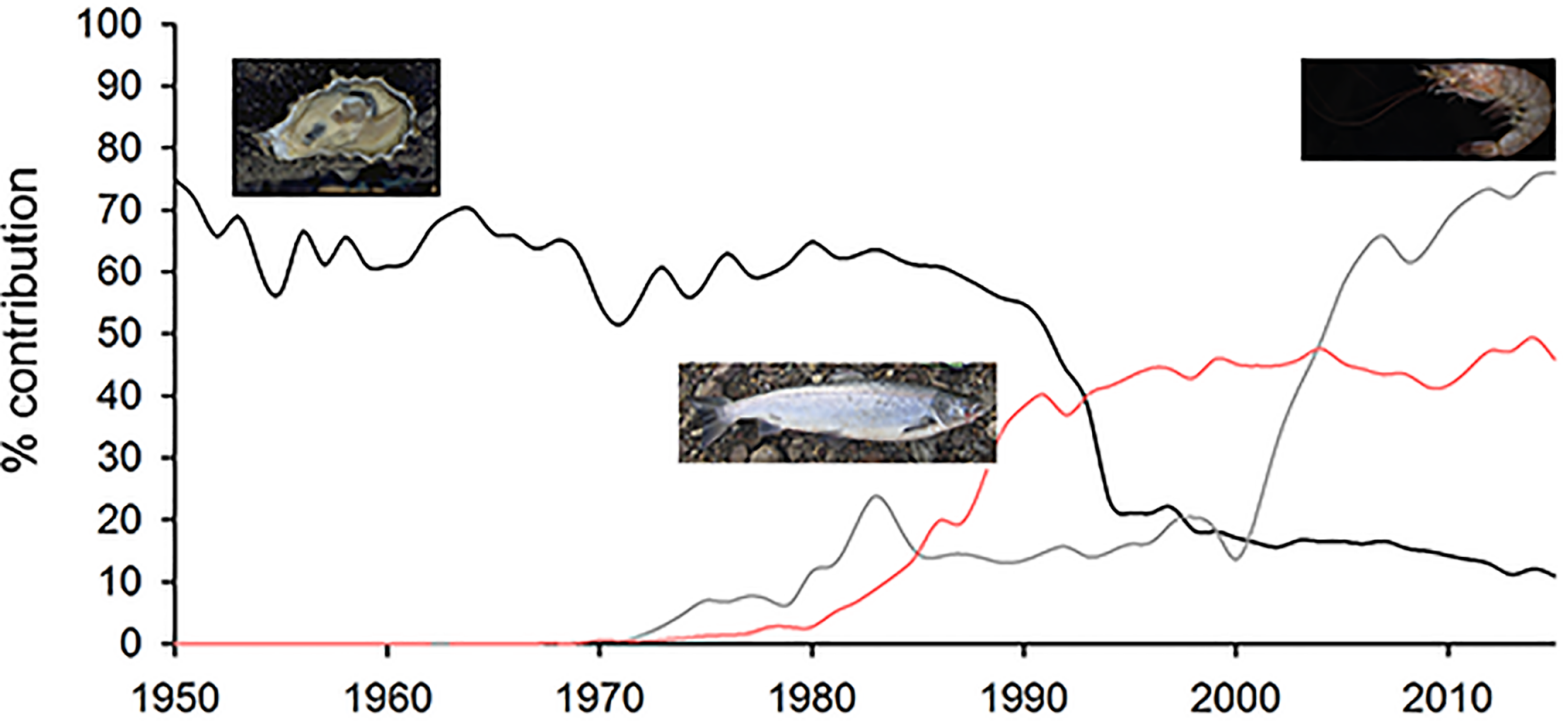
Temporal trends in global aquaculture: % of whiteleg shrimp *Penaeus vannamei* of shrimp and prawn culture in marine and brackish environment, % of Japanese cupped oyster *Crassostrea gigas* of oyster culture in marine environment, and % of Atlantic salmon *Salmo salar* of fish culture in marine environment. Decrease in relative contribution of *C*. *gigas* is related to increase in oyster culture in China, where *C*. *plicatula* and *C*. *rivularis* are cultured on a large commercial scale (Data from [111]). Photo credits: IFREMER (France), Ralf Mae and Nicholas Yap.

The number of species involved and the geographic spread of transplantations appears to have increased in the late 20th century: between 1984 and 1997, 64 countries reported the stocking of 180 species that spend at least part of their life in marine and coastal areas (46 confined to marine environments), although the authors admit these numbers are only a fraction of the global activity [108]. The whiteleg shrimp *Penaeus vannamei*, native to the Pacific coast of Latin America, was introduced widely in the 1970s [92], and constitutes 76% of the world production of cultured penaeids (Fig 2), mainly due to rising production in China and Southeast Asia [71]. In the last decades China has promoted MFE programs [109]. By 2008, over 100 species of finfish, crustaceans, shellfish and jellyfish have been stocked, and almost 20 billion juveniles were released annually [110].

A few species, unintentionally introduced with cultured target species, have been farmed as well. For instance, the seaweed *Undaria pinnatifida* (wakame) was accidentally introduced with *C*. *gigas* into the Mediterranean Sea in 1971 [112,113]. In 1983 it was intentionally transplanted to Brittany, France, for farming, with the risk of its dispersal from the farming sites considered minimal by the French authorities [114]. However, by 1987 reproducing individuals were found on mussel lines next to one farm, and the alga has subsequently spread along the coast from Portugal to the Netherlands [115]. It fouls oyster and mussel lines, aquaculture equipment and boats; massive development may impair aquaculture harvests [116].

Experimental evidence shows that shells of shipped oysters, even if visibly clean, can host a wide range of macroalgal species, including the Japanese seaweed *Sargassum muticum* [117,118], which was introduced to Western Europe in the 1970s with oyster imports [119]. The introduction and rapid expansion of *S*. *muticum* caused one of the most dramatic changes in the vegetation of the upper sublittoral zone, inducing sedimentation, changes in community composition, replacement of native species, and interference with coastal fisheries and recreational activities [120].

The Manila clam *Ruditapes philippinarum*, unintentionally introduced to the North American Pacific coast in the 1930s with Japanese oysters, has become the basis of major mariculture production in the Pacific Northwest [97]. Intentionally introduced in 1983 into the Italian Adriatic to supplement the declining fishery of the indigenous carpet clam *Ruditapes decussatus*, *R*. *philippinarum* ended up supplanting it [98]. Similarly, introduced to the south coast of England for mariculture, *R*. *philippinarum* has spread into the wild providing fishermen with a new crop [99]. Commercial fishing of *R*. *philippinarum* is also very important along the French Atlantic coastline, reaching thousands of tons annually [100].

Concerns about the impact of hatchery fry on wild populations of the same species have been raised since the late 1980s [121]. Of 70 studies which compared hatchery reared and wild stocks, 23 studies showed significant negative effects of hatchery rearing on the fitness of stocked fish, and 28 studies showed reduced genetic variation in hatchery populations [122]. The main concerns are impacts on wild populations such as changes in genetic composition and structure, breakdown of genetic adaptations and loss of genetic diversity [123–125].

Disease agents detrimental to the cultured stocks, associated with the target species, have been of particular concern to the stakeholders for a long time. Some of the early examples include the loss of income following large-scale disease epidemics and mass mortalities of commercially important molluscs infected by introduced “protozoans” (e.g., *Haplosporidium nelsoni* [= *Minchinia nelsoni*]) depressing the mollusc production in Chesapeake and Delaware Bays since the late 1950s [126], and *Bonamia ostreae* affecting *Ostrea edulis* in European waters [127]. These occurrences prompted policymakers and stakeholders to start establishing regulations to limit disease spread and prevent pathogen introductions (see Global policy and legislation).

Extreme weather events may be expected to escalate in intensity and frequency with climate change. Such events play a role in release of NIS from marine as well as land-based mariculture farms and holding pens and causing possible impacts on wild populations [128–130].

#### Live seafood and bait

Humans have moved living species for food and other purposes for a long time (e.g., the 10,000 years timeline from pre-domestication cultivation has been well studied [131]. However, little is known about the historical movement of live edible marine species (see above).

The development of fast, reliable refrigerated transportation for valuable perishable cargo brought about the expansion of a retail market for live seafood around the globe. This has resulted in large amounts of live fish, shellfish and algae being transported and occasionally dumped or released, accidentally or intentionally. Still, live marine seafood trade has received limited attention as a vector of introduction [132]. Information concerning intentional transportation of live marine organisms for consumption is rare until the 19^th^ century, when fast transport, refrigeration and growing affluence provided the means for a global marketplace in live seafood. The American oyster *C*. *virginica*, native to the North Atlantic, was likely the first commercial success of the long-distance live marine seafood trade. As the supply of European indigenous oysters had greatly fallen off due to overharvesting, oysters were shipped from New York to Europe, where they were evidently greatly appreciated: 5000 barrels a week of live oysters packed in flour were shipped in 1882 from New York alone [133,134]. The oysters were shipped live in North America “*as far as railroads and careful packing could get oysters without spoilage*” throughout the 19^th^ and early 20^th^ century [135]. The completion of the transcontinental Central Pacific Railroad in 1869 and the expansion of the ice industry in the late 1800s made possible shipping fresh oysters from the USA East coast to California and eventually as far north as British Columbia [81,136]. The eastern oyster trade is thought to be responsible for a significant percentage of Western Atlantic invaders in San Francisco Bay [137]. Many species of estuarine mollusks, polychaetes, bryozoans, and crustaceans, for example, were inadvertently but successfully introduced with live oyster shipments from the Western Atlantic to the Eastern Pacific [138]. Long after these introductions, other Northwest Atlantic species arrived with a vector that did not exist in the 19th century: live marine worm bait wrapped in seaweed dunnage, the latter hosting many associated species. By this means both the European green crab *C*. *maenas* and the rock periwinkle *L*. *saxatilis* were added to the North American Pacific coast fauna [139,140]. More broadly, the live marine bait trait represents another live trade vector that can transport diverse species to potentially many global regions [141].

Evidence is scant of marine species that have been transported live for the seafood and bait trade and eventually established in the wild. Indeed, only a small number of live imported seafood organisms end up in an environment suitable for their survival. American lobsters, *Homarus americanus*, some with their claws still bound with rubber bands, have been reported from the wild in a number of European countries. Their presence raised concerns about disease transfer, ecological interactions and hybridization with the European lobster, *H*. *gammarus* [142,143]. However, and despite the request, *H*. *americanus*, was not included into the list of invasive alien species of European Union (EU) concern [144]. If numbers of released/discarded organisms are large enough, or if an asexually reproducing organism is released frequently enough, the risk of establishment can increase [145]. Cecere et al. [146] highlight the disregard for regulations concerning storage and handling of imported live seafood and the risk from live seafood organisms held in water in holding facilities and quayside jettisoned discards. While few regulations exist for live bait trade, various studies have explored both the potential importance and possible management strategies [147].

Income and population growth are shifting the live seafood trade from developed to developing countries (China, Southeast Asia), while improvements in chilled cargo shipping and air cargo sustain the emergent long distance live seafood trade patterns [148]. High volumes of lightly regulated transshipment, storage and handling of live organisms pose a clear bioinvasion risk.

#### Ornamental

The horseshoe crab *Limulus polyphemus* is the earliest (1866) marine species considered to have been transported from the United States to Europe as a consequence of the ornamental trade (Table 3; [149]). The aquarium trade vector gained notoriety following the highly-publicized introduction of the seaweed *Caulerpa taxifolia* into the Mediterranean Sea in 1984 [150]. DNA fingerprinting linked the introduction of this invasive alga to public aquaria in Europe [151]. It was established successfully in the Mediterranean Sea and has proven highly disruptive [152], but was eradicated in California, USA [153], and failed to establish in Japan [154,155]. The report of accidental release of lionfish due to a breakage of a large aquarium by Hurricane Andrew is probably erroneous [156], but their subsequent spread across the Atlantic seaboard created a media storm and increased the scrutiny of the ornamental trade as a marine vector [157–159].

**Table 3:** Examples of first records of non-indigenous marine species attributed to the ornamental trade vector. First record is the date of reported collection. “Status” indicates whether species has established self-sustaining populations. “Certainty” refers to confidence of vector assignment; “possible” indicates ornamental as one of several possible vectors, “probable” indicates most likely or sole vector ascribed in reference(s), “certain” indicates a verified aquarium release.

First record	Species	Marine realm	Country	Status, vector certainty	Native realm	Reference
1866	*Limulus polyphemus* (Horseshoe crab)	Temperate Northern Atlantic	Germany	failed; probable	west Temperate Northern Atlantic	[149,160]
1969	*Poecilia latipinna* (Sailfin molly)	Central Indo-Pacific	Australia	established; possible	east North America	[161]
1984	*Caulerpa taxifolia* (Australian green algae)	Mediterranean Sea	Monaco	established; certain	circumtropical to temperate Australasia	[151]
1985	*Pterois volitans* (Red lionfish)	Tropical Atlantic	USA, Florida	established; probable	Indo-Pacific	[158]
1994	*Cromileptes altivelis* (Humpback grouper)	Tropical Atlantic	USA, Florida	established; probable	Western/Central Indo-Pacific	[162]
1995	*Etroplus suratensis* (Pearlspot)	Central Indo-Pacific	Singapore	established; probable	Western Indo-Pacific	[163]
2007	*Scatophagus argus* (Spotted scat)	Mediterranean Sea	Malta	established; probable	Indo West Pacific	[164]
2011	*Acanthurus coeruleus* (Blue tang surgeonfish)	Mediterranean Sea	Cyprus	failed; probable	Tropical Atlantic	[165]
2015	*Zebrasoma xanthurum* (Yellowtail tang)	Mediterranean Sea	Italy	failed, probable	Western Indian Ocean	[166]

Records of marine NIS attributed probably or possibly to the ornamental vector have proliferated in recent decades, although this is likely an underestimate given the lack of marine vector information, let alone ornamental, from many regions [167,168]. However, few of those have established free-living populations (e.g., [157,169,170]).

The marine aquaria trade supplying home and public aquaria has grown into a global industry since the 2000s. The United States and the European Union constitute the largest markets, although trade in Japan, China and Southeast Asia is increasing. The number of marine fish species traded in the US has increased from 1000 in 2001 and 1471 in 2005, to about 2300 in 2011, in addition to 725 invertebrate species [171,172]. Despite the high numbers of species and individuals traded [173], due to its late emergence, the largely tropical origin of the species, and rare instances of release into the sea, few introductions have been attributed to the ornamental trade vector. Increasing trade volumes and global climate change may increase establishment rates for ornamental species introduced to coastal regions of importing countries.

### Maritime canals

The first navigable canal was constructed in the 6^th^ century BCE to join the Mediterranean Sea with the Red Sea by way of the Nile ([174]; Table 4). In the 19^th^ century the same purpose was achieved by excavation of a canal through the Isthmus of Suez. This was followed by another monumental interoceanic canal excavated through the Isthmus of Panama. By breaching natural barriers to the dispersal of marine organisms and altering shipping routes, the interoceanic canals have provided marine biota with new opportunities for dispersal by natural means as well as by shipping.

**Table 4:** Examples of canals connecting different seas (data from [174–177]).

Canal name	Opened	Comments
**Mediterranean and Red Seas**		
	6^th^ century BCE	by way of the Nile
Suez Canal	1869	Cross section area increased from initial 300 m^2^ to 5200 m^2^
**Ponto-Caspian and Baltic Seas**		
Oginskij Canal	1768	This and below: riverine canals
Bug-Pripet Canal	1775	
Mariinskij Waterway	1810	
Severo-Dvinskiy Waterway	1829	
Volga-Don Canal	1952	
**Baltic and North Seas**		
Kiel Canal	1895	First inland waterway in the region in 1398
**Pacific and Atlantic Oceans**		
Panama Canal	1914	Proposed in 1534
Nicaragua Canal		Under consideration

Prior to the opening of the Suez Canal the French malacologist Vaillant [178] had already argued that cutting through the Isthmus of Suez offered an opportunity to examine the immigration of species and the mix of faunas. Within a decade of its opening, two Red Sea bivalves, the Gulf pearl oyster, *Pinctada imbricata radiata* and the mussel *Brachidontes pharaonis* were collected in the Port of Alexandria and Port Said respectively (as *Malaegrina* sp., and *Mytilus variabilis*); the former was already abundant in the port by 1874 and sold in the market [179]. Erythraean biota may traverse the canal by “natural” dispersal, by autonomous active or passive larval or adult movements, but the fouling habits of both bivalve species and their early finding in ports incline us to assume they were vessel-transported. Indeed, Fox [180] observed that fouled tugs and barges employed in the Canal could transport biota from one end to the other. Bivalves are uniquely suited to withstand temperature, salinity and desiccation stress, therefore it was to be expected they would successfully traverse the hypersaline Bitter lakes that served as a salinity barrier in the first decades of the Suez Canal's existence [181]. Successive enlargements of the canal (from 1962 to 2014 its depth increased from 15.5 to 24 m, and its cross-sectional area from 1800 to 5200 m^2^; [182]), combined with the decline of a hypersaline barrier (through dilution), permitted passage to ever larger number of propagules, resulting in the establishment of over 400 Erythraean species in the Mediterranean Sea [183].

The Panama Canal serves as a “bridge of water” between the Caribbean and the Pacific side of the isthmus. The earliest and best-known species reported to have traversed the canal and established a population on the opposite coast is the Atlantic tarpon, *Megalops atlanticus*. This fish, known from the eastern and western Atlantic Ocean, was reported from Lake Gatun and Miraflores lakes in 1935 [184], and later from the sea level end of the canal below Miraflores locks [185]. Recently the species was recorded from Pejeperro Lagoon, on the Pacific coast of Costa Rica [186]. Most of the Atlantic biota that has been recorded from the canal reached Miraflores Third Lock lagoon next to the Pacific entrance of the canal, but failed to establish along the Pacific coast [187]. The freshwater Lake Gatún has formed an efficient barrier to the movement of all but the most euryhaline marine species (except, of course, for any species travelling inside vessels in ballast water). Yet, a large number of organisms have undoubtedly been transported by vessels traversing the canal to be introduced elsewhere ([187] and references therein).

The new, 300-kilometre long Nicaragua Canal joining the Pacific and Atlantic oceans intends to compete for interoceanic traffic by servicing ships too big to pass through Panama’s recently expanded canal. At present, financial problems, along with ongoing environmental and engineering reviews, have delayed the project [175].

## Development of methodologies for detection, identification and surveillance

### Field surveys

Major research focus on marine invasions is relatively recent, emerging initially in the 1960s and 1970s in a few regions, such as the Panama Canal, Suez Canal, and the Pacific coast of North America [137,181,188,189], long after these canals and vectors have been in operation. As a result, NIS data varies considerably among geographic regions and taxonomic groups, resulting in significant imbalance among marine taxa in inventories [190,191]. The data in the available **s**yntheses and checklists (see World Register of Introduced Marine Species, WRIMS [192]), is therefore a product of taxonomic studies, museum collections, field surveys and inventories, rather than standardized surveys designed to detect NIS. While these records are invaluable, providing insights into invasion dynamics and vectors, they are “bycatch” data, collected by different methods for diverse goals. The data quality is uneven across geographic regions, time, and taxonomic groups, making it challenging-to-impossible to interpret patterns of invasion with confidence [8,193]. The historical data generally fail to: (a) estimate the full extent (richness) of marine habitats or taxonomic groups, even at one location, or (b) provide comparable estimates of NIS present across locations or time periods [194,195].

Since the 1970s, survey methodologies have been designed and implemented explicitly to detect marine NIS richness and composition (Table 5; [196]). Most of these have focused on bays and estuaries, especially surrounding ports and marinas [197], as well as canals and offshore structures [198–201]). Most surveys were single events, providing a snapshot documentation of particular area/habitat/taxon. The identities and richness of detected NIS depends upon the methodologies (tools, replication, spatial and temporal scales) employed, season, duration and taxonomic expertise (but see [196]). Often smaller organisms (e.g., meiofauna) and plankton are not included.

**Table 5:** Examples of field surveys designed and implemented to detect non-indigenous marine species.

Survey type	Target group	First applied	Examples of later applications
Rapid assessment surveys	Visual scans for target species and qualitative sampling and analysis, to detect NIS in benthic and pelagic habitats [195,196].	Pacific coast of North America, 1976 [137]	US Atlantic and Pacific coasts, England, Scotland, Ireland, and Panama [202–206].
Quantitative port surveys	Sampling benthic, epifaunal, and plankton communities	Australia, 1996 [207]	Many ports in Australia, New Zealand, and other countries, including adoption by the GloBallast Programme of the International Maritime Organization [196]
Quantitative fouling panel surveys	Sampling hard substrate communities	US Pacific and Atlantic coasts, 1999 [195]	At 36 different bays in the continental US, Hawaiian Islands, and Puerto Rico, with additional bays in Australia, Belize, Ecuador, Panama and other countries [208,209], Canada [210] and Portugal [211].

At the present time, baseline data collected by several survey types exist across multiple global regions. Unfortunately, there is no single global standard survey methodology that has been adopted to allow inter-comparisons among regions. However, several survey types have been replicated spatially, providing some opportunities for regional comparisons. While the value of repeated measures and surveillance is widely recognized, both for evaluating management and rapid response to new incursions [193,212], NIS detection programs comprising repeated community-level surveys appear to still be rare [196] and largely in the formative stages [194,213].

### Application of molecular tools

Molecular tools are increasingly argued as instrumental in overcoming the difficulties associated with conventional taxonomic identification approaches—morphological complexities, cryptic life stages, globally declining taxonomic expertise [214–216]—and addressing the urgent need for efficient and timely detection of new incursions and robust identification of suspected NIS.

**Fig 3:**
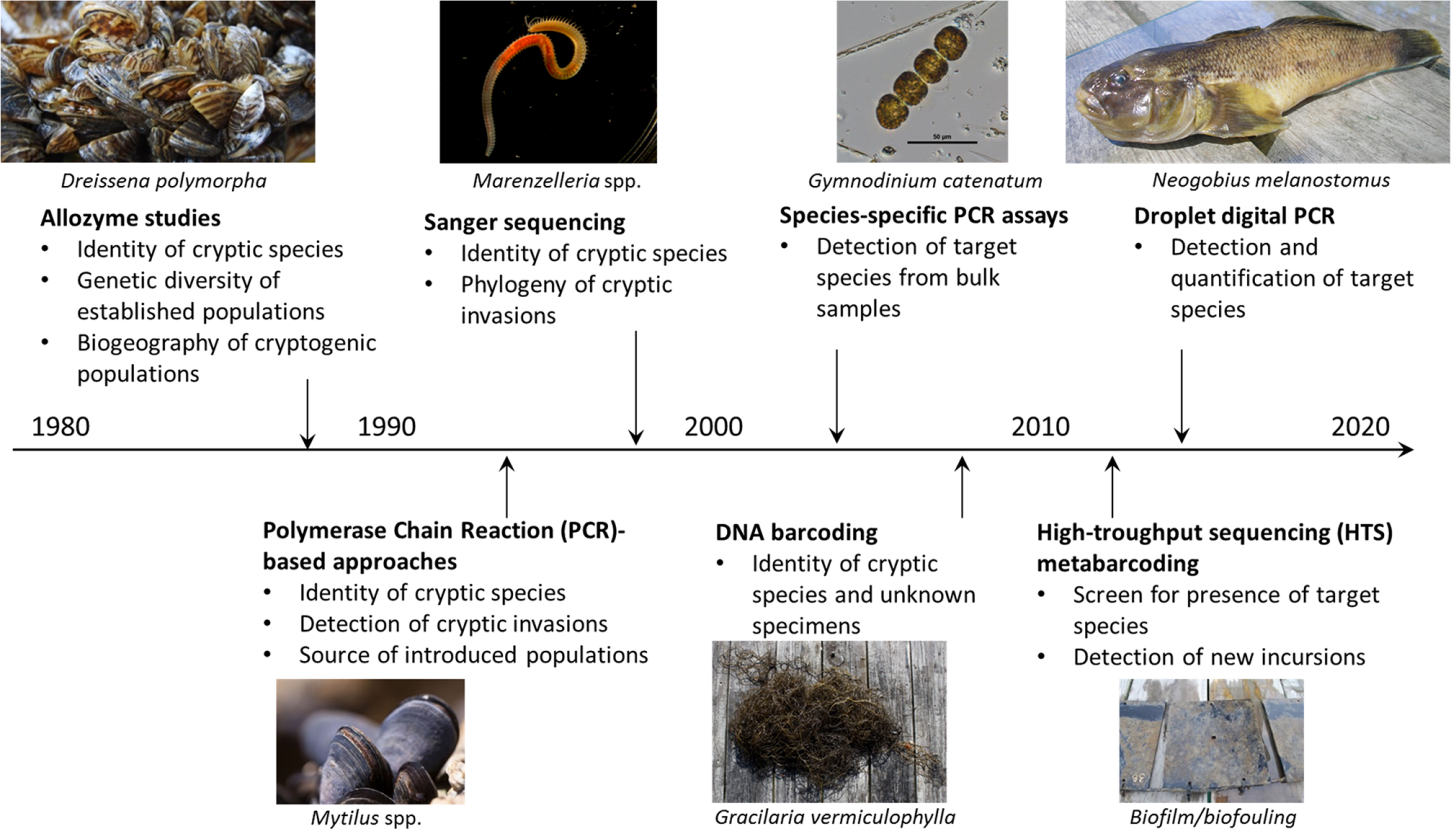
Timeline of molecular methods applications to marine bioinvasions research and surveillance, with images visualising examples of species or biological matrices to which the method was applied in the context of bioinvasions (data from [217,220,221,222,223,225,226,236,238,250]). Photo credits: APRAE SOD (Italy), Jan-Erik Bruun, Vivian Husa, Pixabay, Heli Spilev and Anastasija Zaiko.

The earliest applications of molecular techniques to bioinvasions date to 1980s (Fig 3), when allozyme studies addressed the identity and genetic diversity of *Dreissena* spp. and *Mytilus* spp. [217–219]. Subsequently, DNA-based genetic analyses (e.g., fingerprinting, multilocus genotyping, Sanger sequencing) have been increasingly applied to detect cryptic invasions [220–224].

Molecular techniques facilitate targeted surveillance as species-specific Polymerase Chain Reaction (PCR) and quantitative or real-time (qPCR) assays are cost-efficient tools for biosecurity surveillance, whereby specificity, sensitivity and applicability to environmental DNA (eDNA) enhance scalability of NIS surveillance. In recent decades, an increasing number of species-specific PCR assays have been designed for marine NIS and applied for pre-border [225–227] and post-border [228–231] detection and monitoring.

In early 2000s, a molecular approach to taxonomic diagnosis involving sequencing of short species-specific DNA fragments (DNA barcodes) was introduced to biological research [232]. DNA barcoding has evolved into metabarcoding, allowing potential taxonomic assignment of specimens across entire biotic assemblages [233] from eDNA samples. The uptake of metabarcoding was fostered by the recent development of the High-Throughput Sequencing (HTS) techniques [234,235]. Despite the remaining gaps in understanding the detection limits and quantification capacities of HTS metabarcoding, it is generally recognized as a game-changing approach to environmental surveillance [236–238], including early detection of new incursions, pathway screening, propagule pressure assessment and monitoring of established NIS populations [239–244]).

To date, molecular techniques have been recognized as an important complementary tool for invasion biologists and ecosystem managers [216,245–247]. These methods can provide fast, specific, standardized, high quality and ecosystem-wide information on biodiversity (from microorganisms to macrozoobenthos) and all life stages (including juveniles or larvae–the common spreadable stages of many NIS). The ongoing technological developments and introduction of yet new methods, like shotgun sequencing, digital droplet PCR, gene enrichment techniques and single-molecule sequencers [248–251] make molecular surveillance approaches even more appealing for routine biosecurity applications. Certain caveats remain relative to the specificity of non-target molecular methods (such as metabarcoding), given that reference sequence databases are far from complete and error-free, and truly universal marker genes do not exist yet [58,97,200]. Another shortcoming is the current lack of quantitative capacity, especially when applied to multicellular organisms. As yet robust biodiversity or abundance information required for impact assessments, management and enforcement is unattainable [252]. Taxonomic expertise remains a critical requirement for NIS assessment and management, and the advantages of integrated taxonomic approaches using both molecular and morphology-based methods are repeatedly emphasized by researchers.

### Citizen science

Historically, members of the public have played a key role in detection and surveillance, advancing our understanding of changes in species distributions and abundances through time and across diverse ecosystems and taxonomic groups [253,254]. The valuable contribution of such observations, and their potential as an information resource, have gained increasing recognition over the past decades. This has led to a surge in development of citizen science programs with a diverse range of applications, including the detection and study on NIS in marine systems [254,255].

The discovery of new marine NIS in a region has often been through chance encounter by fishermen, divers, and the public at large–who report novel and conspicuous organisms–providing an informal and diffuse detection network. For example, a fisherman in Chesapeake Bay provided the initial report of the Chinese mitten crab *Eriocheir sinensis* for the Atlantic coast of the United States [256].

The opportunity for “crowdsourcing” NIS detection and surveillance has been greatly enhanced by broad accessibility of new technologies, including the ability to instantly collect and share georeferenced data and photographs through mobile phone and web-based platforms, and also by increased focus and tools for optimizing the structure of citizen science efforts [257–260]. This has led to increasingly organized and formally structured campaigns–from bioblitz activities to sustained detection and monitoring for conspicuous NIS–including those in the marine realm.

The contribution of citizen science programs for NIS detection and surveillance is expected to expand over time, helping to address the limited funding and spatial/temporal coverage available with current programs [261]. Current research is demonstrating the high-quality data possible for particular types of measures and marine taxa [254,255]. There are some constraints that need to be considered in program design and expectations, including selecting large-bodied, conspicuous taxa with easy-to-recognize diagnostic characteristics. In the future, genetic tools may be adopted by citizen science programs to enhance the potential taxonomic scope and validation.

### Post-invasion management

Post-introduction management efforts date back to the mid-20th century if not earlier [262]. Management attempts may be directed at, (1) the *eradication* of small, spatially restricted populations of newly introduced NIS, (2) *reducing the local abundance* of already established NIS, or (3) *preventing their spread*. Williams and Grosholz [263] have summarized nearly 20 examples of successful, unsuccessful, and ongoing eradication programs for introduced estuarine and coastal species from 1951 to 2006. Very few programs result in the permanent removal of NIS.

Efforts that seek ways and methods to control the abundance and spread of abundant pest species continue. Examples include the Asian seaweed *Sargassum horneri* in southern California [264], the grape algae *Caulerpa racemosa* in the Mediterranean Sea [265,266], the Asian ascidian *Didemnum vexillum* in the North Atlantic Ocean [267], and the Indo-Pacific lionfish *Pterois* spp. in the Caribbean Sea [268,269]. We emphasize that prevention through the restriction and reduction of introduction pathways and vectors is the overwhelmingly preferred option, given that management of already established NIS is increasingly viewed as unfeasible and unsustainable (e.g., [194,270]).

## Impacts quantification

One of the earliest quantitative evaluations of ecological impact of NIS dates back to the 1920s, when the Atlantic mussel *Geukensia demissa* endangered the California clapper rail *Rallus obsoletus* in San Francisco Bay. It was estimated that at least 75% of the adult rail and 25% of the chicks were negatively affected [271]. However, only in the late 1970s, with documentation of the increasing domination of non-indigenous biota and associated changes in native biota (e.g., the Baltic Sea [272] and San Francisco Bay [273]), did quantitative evaluation become firmly established (Table 6). The last two decades have substantially increased our knowledge base through experimental and quantitative studies on the impacts of NIS worldwide (Europe, North and South America, South Africa, and Australasia), although the number of studies remains relatively small compared to the number of marine introductions.

**Table 6:** Examples of the ecological and environmental impacts of non-indigenous marine species.

Species and Origin	Introduced location	Impact	Reference(s)
**Chlorophyta (green algae)**			
*Caulerpa taxifolia* (Australian green alga)	Mediterranean Sea	Reduces productivity of two native macrophytes (*Cystoseira barbata f*. *aurantia* and *Gracilaria bursa-pastoris*). Decrease in mean species richness, mean density and mean biomass of fish	[274,275]
*Codium fragile fragile* (Japanese green alga)	USA: New England	Reduces diversity of other seaweeds; impacts shellfish populations; transports large numbers of native slipper limpets (*Crepidula fornicata*) onto shore	[276]
	Canada: Nova Scotia	Competitive advantage over native seaweeds (kelps and other algae) through opportunistic exploitation of disturbed patches in kelp beds; once established as dense meadows, prevents kelp recolonization and persists as the dominant canopy-forming seaweed	[277]
**Tracheophyta (flowering plants)**			
*Zostera japonica* (Japanese eelgrass)	Canada and USA: British Columbia to Oregon	Converted vast areas from open soft-sediment habitat to rooted vegetation, a profound habitat alteration influencing sediment patterns (mean sediment grain size and sediment volatile organics) and resident fauna richness and densities, which alters interactions between pre-existing species	[32,278]
*Spartina alterniflora* (Northwest Atlantic saltmarsh cordgrass)	Argentina and Atlantic coast of South America	Changed previous soft-bottom habitat to coastal marshes, with vast unrecorded and thus overlooked shifts in bird, fish, and invertebrate biodiversity and immense shifts in algal vs. detritus production, with the concomitant trophic cascades	[279]
	USA: California and Washington	Changed sediment dynamics, decrease algal production through shading, loss of shorebird feeding habitat, reduction of shrimp and oyster habitat, altering fish and wildlife habitat	[32]
**Ctenophora (comb jellyfish)**			
*Mnemiopsis leidyi* (West Atlantic comb jelly)	Black Sea	Predation on fish eggs and larvae and their food (zooplankton) in addition to increased nutrients and high fishing pressure caused a collapse of small planktivorous fish	[280,281]
*Beroe ovate* (West Atlantic comb jelly)	Black Sea	Predation combined with seawater warming and decreased fishing pressure, caused a marked decrease in the density of *M*. *leidyi* with concomitant increase in the abundance of zooplankton (about 5-fold) and ichthyoplankton (about 20-fold)	[280,282,283]
**Annelida: Polychaeta (worms)**			
*Ficopomatus enigmaticus* (Australian tubeworm)	Argentina	Reef-building species providing habitat for native species, such as the crab *Cyrtograpsus angulatus*, which dramatically increases and then negatively impacts the abundance of native worms and a major effect on habitat integrity; reefs alter bedload transport and water flow	[284,285]
*Sabella spallanzani* (Mediterranean fan worm)	New Zealand	Dense aggregations significantly alter communities, outcompeting native species for space and food. Form ‘canopies’, affecting the recruitment, survival and growth of other biofouling organisms, by overgrowing and dislodging native taxa	[286–289]
*Marenzelleria* spp. (North American spionid worms)	Baltic Sea	Alters benthic community and nutrient regulation, including enhancing phosphorus flux from sediment to water on a basin-wide scale, potentially countering eutrophication mitigation. Re-oxygenates oxygen depleted deep sediments.	[290,291]
**Mollusca: Gastropoda (snails)**			
*Littorina littorea* (European periwinkle)	USA: New England	Regulate much of intertidal diversity directly or indirectly, including reducing algal diversity and abundance through direct consumption; controls species composition and diversity in tidepools; may impact salt-marsh dynamics by consuming *Spartina* rhizomes; displaces native mudsnail *Tritia obsoleta*, setting upper and lower limits of native's distribution; increased abundance in some regions of native hermit crabs	[32,276,292,293]
*Batillaria attramentaria* (Japanese mudsnail)	USA: California	Competitive displacement of the native mudsnail *Cerithideopsis californica*	[294]
	USA: Washington	Leads to increase abundance of NIS on *Batillaria* shells, of non-indigenous eelgrass, and of native hermit crabs	[295]
*Zeacumantus subcarinatus* (New Zealand rock pool snail)	Australia: Sydney	Competitive displacement of the native rock pool snail *Bembicium nanum*	[296]
*Tritia obsoleta* (= *Ilyanassa obsoleta*) (Atlantic mud snail)	USA California: San Francisco Bay	Competitive displacement of the native Pacific mud snail *Cerithideopsis californica*	[297]
*Rapana venosa* (Japanese rapa whelk)	Black Sea	Significant impact on the native bivalves *Ostrea edulis*, *Pecten ponticus*, and *Mytilus galloprovincialis* due to predation	[298]
	Uruguay: Rio de la Plata estuary	Predominate top-down effect on abundance of most native bivalves	[299]
*Crepidula fornicata* (West Atlantic slipper limpet)	France Atlantic coast: Bay of Saint-Brieuc	Conversion of former soft substrate to hard, shelled substrate, resulting in decreased abundance of certain suprabenthic species (such as mysids)	[300]
	Germany: Wadden Sea	Reduces survival and growth of native mussel *Mytilus edulis*	[301]
	France Atlantic coast: Arcachon Bay	Homogenizes benthic community (decreasing beta-diversity) but increases local diversity (alpha-diversity), which may alter interactions between species	[302]
**Mollusca: Bivalvia (mussels, clams)**			
*Corbula amurensis* (= *Potamocorbula amurensis*) (Asian corbula)	USA California: San Francisco Bay	Seasonal loss of water column productivity, with cascading trophic impacts	[303–305]
		Chronic depression of estuarine copepods that are food of several fish species that are also in decline	[306]
*Arcuatula senhousia* (= *Musculista senhousia*), *Ruditapes philippinarum* (= *Venerupis philippinarum*), *Mya arenaria*, *Gemma gemma* (Japanese mussel, Manila clam, Atlantic softshell clam, and Atlantic gem clam, respectively)	USA California: San Francisco Bay	Control of water column productivity through grazing (filtering)	[307,308]
*Arcuatula senhousia* (= *Musculista senhousia*) (Japanese mussel)	USA California: Mission and San Diego Bays	Intertidal reef-like mussel mats dominate shores, depressing native clam and seagrass populations	[309–311]
	New Zealand: Auckland region	Decline in infaunal bivalves	[312]
*Mytilus galloprovincialis* (Mediterranean mussel) and *Semimytilus algosus*(Pacific mussel)	South Africa	Now, alien mussels and barnacles (e.g. *Balanus glandula*) dominate on some wave-swept shores, but see [314] for changes to habitat complexity and abundance of both native and introduced species following sequential invasions of rocky shores on Marcus Island on west coast	[313,314]
*Mytilus galloprovincialis* (Mediterranean mussel)	South Africa	Competitive exclusion of indigenous mussel *Aulacomya ater* and large limpets; enhancement of recruitment of juvenile limpets and increased habitat availability for mussel infauna	[315]
	New Zealand	Competitive domination in subtidal benthic community	[316]
	California	Replaced native mussel *Mytilus trossulus*	[317]
**Arthropoda: Crustacea: Isopoda (pill bugs)**			
*Sphaeroma quoianum* (New Zealand burrowing isopod)	USA California: San Francisco Bay	Severely erodes marsh and peat-bank edges	[32]
	USA: Oregon to California	Major intertidal bioeroder, damaging and destabilizing marsh banks, friable rock, and polystyrene marine floats (for the latter, leading to production of fine plastic dust, exacerbating plastic pollution in the ocean)	[318]
*Sphaeroma terebrans* (Indian Ocean boring isopod)	USA: Florida mangroves	Bores into and destroys mangrove (*Rhizophora mangle*) prop roots	[32]
**Arthropoda: Crustacea: Amphipoda (amphipods)**			
*Corophium volutator* (European amphipod)	Atlantic North America: Bay of Fundy	Significant ecosystem engineer and often major prey of migratory birds; long overlooked as an invasion	[319]
**Arthropoda: Crustacea: Decapoda (crabs)**			
*Carcinus maenas* (European green crab)	USA: New England	Alters diversity and abundance of many native prey species; alters abundance and morphology (phenotypes) of native intertidal snails; precipitous declines in native soft-shell clam *Mya arenaria*	[276]
	Canada Atlantic coast	Significantly alters mud-bottom community structure through habitat disruption	[320]
*Hemigrapsus sanguineus* (Asian shore crab)	USA: New England	The most abundant crab on many intertidal shores, leading to significant declines in abundance of other crabs, snails, mussels, barnacles, and many other species	[321,322]
**Arthropoda: Crustacea: Stomatopoda (mantis shrimps)**			
*Gonodactylaceus falcatus* (Indo-Pacific mantis shrimp)	USA: Hawaiian Islands	Competitive displacement of native mantis shrimp *Pseudosquilla ciliata*	[323]
**Bryozoa (moss animals)**			
*Membranipora membranacea* (European bryozoan)	Canada: Nova Scotia	Significant loss of native seaweeds due to epibiotic colonization of blades	[277,324]
*Tricellaria inopinata* (Pacific Bryozoan)	Italy: Northern Adriatic Sea	Significant loss of a highly diverse native bryozoan community	[325]
**Echinodermata (sea stars)**			
*Asterias amurensis* (Japanese sea star)	Australia	A major predator and a keystone species exerting top-down control of its prey, especially native bivalve populations; caused local extinctions of several species; long-term decline of certain demersal fish due to competition with *Asterias*	[326–328]
**Chordata: Ascidiacea (sea squirts)**			
*Didemnum vexillum* (Japanese compound sea squirt)	USA: New England, Georges Bank	The key driver of biodiversity decline in the epibenthos, restructuring invertebrate community	[329]
*Clavelina oblonga* (Caribbean sea squirt)	USA: North Carolina	Dominates fouling community with significant declines in biodiversity	[330]
*Ciona robusta* (Japanese Ciona sea squirt)	USA California: San Francisco Bay	Significantly depresses biofouling community species richness	[331]
**Teleostei (fish)**			
*Siganus rivulatus* and *Siganus luridus* (Red Sea rabbitfish)	Mediterranean Sea: Levant, Aegean Sea	Replaces canopy-forming algae with ‘barrens,’ causing reduction in biogenic habitat complexity, biodiversity and biomass	[332,333]
*Pterois volitans* and *Pterois miles* (Indo-Pacific lionfish)	Caribbean Sea	Predation caused a 95% decrease in abundance of small reef fish at some invaded sites and a 65% decline in native fish biomass on heavily invaded reefs; concomitant cascading effects on reef food webs and benthic community structure, including altering balance of competition between native coral reef fish	[334–339]

The first attempts to actually define, evaluate and compare measures of impact in a comprehensive manner started in the late 1990s, when recommendations were made on how the field of invasion biology might proceed in order to build a general framework for understanding and predicting impacts [340]. The first comprehensive ecological impact assessment study was conducted by Ruiz et al. [341], who analysed the reported ecological impacts of 196 species in the Chesapeake Bay through incorporation of various types of information (such as the impact type, information type and the effect of magnitude into the analysis). The more integrated impact evaluation framework–BINPAS (Biological Invasion Impact / Biopollution Assessment System)–to translate the existing data on invasive alien species impacts into uniform biopollution measurement units was developed in the 2000s [342]. Perhaps the most comprehensive and inclusive, but very data-hungry NIS introduction consequence (impacts) matrix has been developed by Hewitt et al. [343], where impacts are assessed against eleven value sets (habitat and habitat forming species, biodiversity, trophic interactions, nationally important and ecologically valuable species, assets (places) of environmental significance, economic values, social values, cultural values, national image (iconic places or species), aesthetic values and human health at a 5-grade level (from negligible to very low to extreme). During the last decade, a few additional impact evaluation frameworks, with inclusion of both qualitative and quantitative data, and ecological and socioeconomic information, were proposed (e.g., [344–349]). However, and despite pilot evaluations, none of them have proven so far robust enough to be able to reach the status of wide cross-regional applications in the marine realm.

## Known/unknown/unknowable–some long-standing dilemmas

### Implications of overlooked invasions

If between 1500 and 1800 only three marine species a year were successfully introduced but undetected as such around the world, “*then nearly 1*,*000 coastal species of marine organisms that are now regarded as naturally cosmopolitan are in fact simply early introductions*” [350]. These were referred to as the “Missing 1000” [351]. The estimate may be far too low, given that international shipping had commenced within ocean basins more than 2000 years ago and that more than 200 years have passed since 1800. Overlooked invasions may have profoundly altered the structure and function of pre-existing marine communities, which have long been studied as if they resulted from long term evolutionary processes. This phenomenon was referred to as “*ecological mirages*: *illusions that have seriously hampered our ability to recognize the nature of pre-existing native ecosystems*” [34]. Some examples include the wood-boring isopod *Sphaeroma terebrans*, and the stoloniferous fouling bryozoan *Amathia verticillata*. The isopod *S*. *terebrans* was transported by ships prior to the 1860s from the Indian Ocean to the Western Atlantic, where it altered the mangrove forest communities over a vast area, and yet their remarkable ecological consequences have been rarely noted [352]. The bryozoan *Amathia verticillata* (“zoobotryon”) occurs worldwide in tropical and warm-temperate waters, mostly in ports and marinas, or anthropogenically altered areas such as shellfish farming bays and lagoons [353]. Although long considered native to the Mediterranean Sea, it may be native to the Caribbean Sea and introduced elsewhere [354].

### Non-indigenous vs. cryptogenic species

Species that we are unable to determine as to whether they are native or non-indigenous are termed cryptogenic [93]. The failure in classification may be due to their early introduction/establishment, misinterpretation due to systematics (pseudoindigenous species, imperfect or low-resolution taxonomy); complex biogeographic and community histories (widespread intraoceanic and interoceanic corridor species, neritic species with presumptive oceanic dispersal); or sampling (unexplored or little known habitats or communities, small population sizes) [12]. Even widely distributed, seemingly well-known species are prone to these issues. For example, the mussel *Mytilus galloprovincialis*, native to the Mediterranean Sea, was mistakenly re-described as a native species following introduction (e.g., re-described as *M*. *diegensis* in California, and *M*. *planulatus* in Australia [14]). Similarly, the "endangered" European seaslug *Corambe batava* was eventually recognized as the common American seaslug *C*. *obscura*, but only 125 years after it had been described [12]. Resolution of cryptogenic status [355,356] relies greatly on data availability and molecular tools, and is therefore a subject for continuous improvement and change. Similarly, the sea squirt *Ciona intestinalis* was recently recognized as comprising two species, both introduced elsewhere, one widely [357–359].

### Certainty in introduction pathways

Vectors of introduction are known with high certainty only for a selected group of NIS (i.e., documented deliberate introductions, or where linkage between donor/regional regions, life history, and historical records point to a sole possible vector). Establishing the vector of introduction for the majority of NIS is still largely a matter of inference rather than evidence. Vectors are deduced from biological and ecological traits of the species, the habitats they occupy in the native and introduced range, the timing of first record, e.g. before or after the advent of ballast water use (see Modern shipping), relative to regional trade patterns and vector activity, e.g. mariculture or shipping [9,356,360]. Nevertheless, many NIS display traits and habitat preferences that may give a good reason to expect association with multiple vectors, e.g. NIS commonly found in harbours may have been introduced by ships in fouling or in ballast [361]. The compilation of regional inventories of marine NIS in the 1990s supplied the impetus for discussion of vectors. Carlton and Ruiz [362] provided terminology (polyvectic, cryptovectic) and a conceptual framework for marine bioinvasion vectors that distinguished cause, route, and vector for an invasion, as well as a vector's tempo, biota and strength.

Despite a burgeoning interest in invasion science in the last 25 years, a surprising number of gaps exists in our knowledge and understanding of how vectors operate. It is widely accepted that “*the detailed invasion history of most species*, *which may include multiple introductions via multiple pathways*, *will never be known with absolute certainty*” [360]. Over the past 20 years, designation of vector probability has been discussed (see Table 7 for a classification and examples). Most authors prefer the multiple vectors scheme, which allows for a range of possible introduction scenarios, and can be weighted depending on probability/certainty, or simply accorded equal value (as in most literature). No consensus has been reached on the optimal strategy to deal with the vexing issue of vector uncertainty.

**Table 7:** Schemes describing vector uncertainty in marine bioinvasions.

Scheme	Region	Reference
Single vector: each species assigned only to its most-likely vector	Britain	[363]
	Baltic Sea	[364]
	Mediterranean Sea	[346]
Multiple vectors (A): each species assigned to one or more distinct vectors, all equally probable	USA: California	[361,365]
	South Africa	[366]
	Great Britain	[367]
	Malta	[368]
	Portugal	[369]
Multiple vectors (B): each species assigned to one or more distinct vectors, each vector scored in accordance with its probability	Australia: Port Phillip Bay	[356]
Multiple vectors (C): each species assigned to one or more distinct vectors, each vector assigned certainty value	USA: Puget Sound	[202]
	Baltic Sea	[370]
Multiple vectors (D): each species assigned to one vector category; some categories are polyvectic (*e*.*g*. “Culture + Vessels”)	USA, Canada: northern California to British Columbia	[360]
	Europe	[9]
	Mediterranean Sea	[183]

## Perceptions of marine bioinvasions

In interpreting historical processes, one is aware of the influence of the societal drivers underpinning human perceptions and actions and how these change over time. Introduction of marine NIS was not widely considered a potential threat until the early 1980s. Since then, increasing evidence of the impacts of marine NIS (see Impacts quantification) has helped raise public awareness and altered community perception of marine introductions, followed by a growing realization that the “shifting baselines” syndrome [10] applies to introductions as it does to fisheries. Although invasion scientists provide ever more evidences of socio-economic and ecological impacts of marine bioinvasions (which are context dependent, i.e., not all NIS manifest the same level of environmental, economic, societal and other impacts, and these may vary over time and space; further, some NIS with known ecological impacts may also be considered to impart ecological or socioeconomic advantage [371–373]), the discipline has elicited criticism and has been intensely disputed [374,375].

Public awareness and perceptions, driven by environmental, economic and social consequences of invasive NIS, can determine the level of support for policy and management actions used to control/manage (potentially) harmful NIS. Unlike terrestrial and inland aquatic bioinvasions, quantitative data or assessments for impacts for most marine NIS are scarce. This is a “catch-22” situation–the impacts for the vast majority of marine NIS remain unknown for want of funding, which depends on public support, which in turn is decided according to public concerns and priorities. “*Unless impacts are conspicuous*, *induce direct economic cost*, *or impinge on human welfare*, *they fail to arouse public awareness*” [270]. Indeed, media recently scanned for coverage of NIS introductions to the Mediterranean Sea, highlighted species considered human health hazards rather than those of high ecological risk [376].

Despite evidence of major irreversible ecological impacts by many NIS and some shift in societal perceptions, NIS are not yet at the forefront in marine management. An online survey of more than 10,000 respondents from 10 European nations examined “*the public’s informedness and concern regarding marine impacts …and priorities for policy and funding*” revealed that respondents were the least informed on NIS issues and prioritized marine invasive species at the bottom of research funding needs [377]. The same attitude is apparent even amongst marine conservationists. A recent literature review found that biological invasions are being widely disregarded when planning for conservation in the marine environment; of 119 articles on marine spatial plans in the Mediterranean Sea, only three (2.5%) explicitly took NIS and marine bioinvasions into account [378], even in the NIS-beset Levantine Basin [379].

## Policy and legislation: Honored in the breach

### Global policy and legislation

As NIS are often introduced or spread by global transport and trade and just as often have transboundary impacts, their prevention and management is an international issue requiring global policy. To date, only two global instruments are strictly legally binding.

The United Nations Convention on the Law of the Sea (UNCLOS) is the first global legally binding legislation to deliver a clear message: “*States shall take all measures necessary to prevent*, *reduce and control … the intentional or accidental introduction of species*, *alien or new*, *to a particular part of the marine environment*, *which may cause significant and harmful changes thereto*.” [380]. Considering the negative environmental effects of intentional and unintentional introductions into the marine environment, the uncertainty as to which of the present and continually introduced NIS will have an impact and at what scale, the unfeasibility of eradication and restoration and vectors’ build-up (e.g., commercial and recreational maritime transport, mariculture, canals), one would expect decision makers to follow UNCLOS and adopt a preventive and precautionary, if not environmentally-focused approach. Disappointingly, examination of policy and legislation actions reveals reactive, piecemeal development, often following disastrous and costly NIS outbreaks.

Article 8(h) of the Convention on Biological Diversity (CBD) requires Parties, as possible and as appropriate “*to prevent the introduction of*, *control or eradicate those alien species which threaten ecosystems*, *habitats or species*” [381]. A decade after the adoption of the CBD, noting “… *that there are certain gaps and inconsistencies in the international regulatory framework from the perspective of the threats of invasive alien species to biological diversity*”, the Conference of the Parties adopted the ‘Guiding Principles for the Prevention, Introduction, and Mitigation of Impacts of Alien Species That Threaten Ecosystems, Habitats, or Species’ and urged the development of national and regional invasive species strategies and action plans [382]. The revised Strategic Plan for 2011–2020 adopted by the CBD in 2010, supported by 20 “Aichi Biodiversity Targets”, states “*By 2020*, *invasive alien species and pathways are identified and prioritized*, *priority species are controlled or eradicated*, *and measures are in place to manage pathways to prevent their introduction and establishment*.” [383]. 2020 will now pass without these targets achieved, and they remain a major challenge.

After establishing the Working Group on the Introduction and Transfers of Marine Organisms (WGITMO), the International Council for the Exploration of the Sea (ICES) adopted the first version of what was to become an internationally recognized Code of Practice on the movement and translocation of non-native species for fisheries enhancement and mariculture purposes. The Code contained two recommended procedures: i) for all species prior to reaching a decision regarding new introductions, and ii) for introductions or transfers which are part of current commercial practice [384]. The Code of Conduct for Responsible Fisheries, promulgated by the Food and Agriculture Organization (FAO) of the United Nations, based on ICES’ Code of Practice, includes recommendations concerning non-indigenous aquaculture species [385]. Article 9.3.1 urges “… *efforts should be undertaken to minimize the harmful effects of introducing non-native species … especially where there is a significant potential for the spread of such non-native species … into waters under the jurisdiction of other States as well as waters under the jurisdiction of the State of origin*. *States should*, *whenever possible*, *promote steps to minimize adverse … effects of escaped farmed fish on wild stocks*”. Although widely endorsed, few people report applying its principles [386]. Further recommendations as to management and disease surveillance and notification have developed into a comprehensive Aquatic Animal Health Code [387,388]. However, the legislation is primarily focused on the economic issues, by stating: “*The principal aim of the International Aquatic Animal Health Code…*. *is to facilitate international trade in aquatic animals and aquatic animal products*. *The International Aquatic Animal Health Code… attempts to achieve this aim by providing detailed definitions of minimum health guarantees to be required of trading partners in order to avoid the risk of spreading aquatic animal diseases*.” [387]. The industry’s precautionary principle does not extend to feral introduced shellfish and fish, nor the many non-pathogenic organisms introduced with the target species. In 2006, considerations and suggestions to be taken into account by decision makers and managers when using–or deciding on the use of–NIS for aquaculture purposes were developed under the IUCN umbrella [389].

In response to national concerns, the US Congress passed the “Non-indigenous Aquatic Nuisance Prevention and Control Act” in 1990, and the Commonwealth Government of Australia, Australian Quarantine and Inspection Service, introduced voluntary ballast water guidelines for ships entering Australian ports from overseas. The guidelines developed under both the US and Australian initiatives were adopted the next year by IMO’s Marine Environment Protection Committee (MEPC) as the “International Guidelines for preventing the introduction of unwanted aquatic organisms and pathogens from ships' ballast water and sediment discharges” [390], and adopted by the IMO Assembly in 1993 [391]. A few years later, “Guidelines for Control and Management of Ships’ Ballast Water to Minimize the Transfer of Harmful Aquatic Organisms and Pathogens” were published [392]. The second legally binding instrument is the “International Convention for the Control and Management of Ships' Ballast Water and Sediments” (BWMC), which is directed at managing the discharge of ballast water and sediments through ballast water exchange and treatment [393]. The BWMC sets a global standard for the minimum amount and size of organisms permissible in ballast water discharged by ships. The BWMC formally entered into force in September 2017. However, on July 2017, the MEPC accepted an amended implementation scheme for ships to comply with the D-2 biological standard and set new schedules for ship owners to meet the requirements for ballast water treatment, in some cases delaying by two years the deadlines for installing those systems on ships already in operation. The deadline for mandatory installation of an approved BWMC system is now, in some cases, as late as 2024, twenty years after adoption of BWMC [394].

Vessel biofouling, a major vector in the translocation of NIS (see Modern shipping), was for many decades, and still is, held in partial check by the application of toxic paints. In 2001 IMO adopted the “International Convention on the Control of Harmful Anti-fouling Systems on Ships” [395], which entered into force in 2008, following studies that attributed the failure of some oyster culture operations and severe pathological conditions in some marine organisms to leachate from antifouling paints, particularly tributyltins (TBT). Concerns over the surge of vessel biofouling moved IMO to adopt voluntary “Guidelines for the control and management of ships' biofouling to minimize the transfer of invasive aquatic species” [396], followed by approving “Guidelines for the control and management of ships’ biofouling to minimize the transfer of invasive aquatic species” [397].

### European Union policy and legislation

The European Union (EU) has a substantial body of environmental laws. Its biodiversity legislation, most notably the Habitats Directive, forms the cornerstone of Europe's nature conservation policy [398]. Article 22(b) states that in implementing the provisions of this Directive, Member States shall “*ensure that the deliberate introduction into the wild of any species which is not native to their territory is regulated so as not to prejudice natural habitats within their natural range or the wild native fauna and flora*” [398].

The “Convention on the Conservation of European Wildlife and Natural Habitats” (Bern Convention) requires Contracting Parties “*to strictly control the introduction of non-native species*” [399]. In 1984 the Committee of Ministers concerning the introduction of non-native species recommended that “*the governments of the member states prohibit the introduction of non-native species into the natural environment*” (with exceptions following risk assessment), “*take the necessary steps to prevent as far as possible the accidental introduction of non-native species*, *and inform governments of neighboring countries concerned of introduction schemes or accidental introductions*” [400]. However, with the single exception of controlling proliferation of *C*. *taxifolia* in the Mediterranean Sea, these recommendations concern terrestrial and inland waters [401].

Some preventive measures to curb introductions of NIS with cultured organisms were initiated in Europe (France) as early as the 1930s, with a state decree limiting oyster transfer due to the concomitant occurrence of the American slipper limpet *Crepidula fornicata* [402]. Also, brood stock of *C*. *gigas* imported to France from Canada and Japan underwent in the 1970s at the customs clearance “*histological analysis*, *presence of predators and commensal species… spat were immersed in freshwater to destroy fouling organisms and predators*” [403]; despite this, the authors acknowledge that a long list of “*concomitant exotic species*” were still introduced. Building on the ICES Code of Practice (see 7.1), the European Community (EC) adopted in 2007 a regulation concerning use of alien and locally absent species in aquaculture, reasoning that as “*Aquaculture is a fast-growing sector… it is important for the aquaculture industry to diversify the species reared*” [404]. The policy objective, developed to control new intentional introductions *“… is to optimise benefits associated with introductions and translocations while at the same time avoiding alterations to ecosystems*, *preventing negative biological interaction*, *including genetic change*, *with indigenous populations and restricting the spread of non-target species and detrimental impacts on natural habitats*.” [404]. Yet records of culture-transported NIS established in the wild–including macrophytes, molluscs, crustaceans–continue unabated [405–410].

The EU Marine Strategy Framework Directive (MSFD) aims to protect the marine environment by achieving “Good Environmental Status” (GES) in European Seas by 2020 [5,411], a target again no longer feasible. It comprises an explicit regulatory objective “*Descriptor 2*: *Non-indigenous species introduced by human activities are at levels that do not adversely alter the ecosystems*.” A recent report assessing Member States’ monitoring programmes found low adequacy and compliance for Descriptor 2, as only 5% of the monitoring activities were linked with NIS, and warned that “*Monitoring programmes for NIS will require a clear acceleration to ensure proper coverage given the MSFD deadlines for the update of marine strategies by 2018*, *and achieving GES by 2020*” [412]. A later, even less sanguine document “…*highlighted that more efforts were urgently needed if Member States are to reach good environmental status by 2020*. *The results showed the necessity to significantly improve the quality and coherence of the determination of good environmental status by the Member States*. *In addition*, *the assessment recognised that regional cooperation must be at the very heart of the implementation of Directive 2008/56/EC*. *It also emphasised the need for Member States to more systematically build upon standards stemming from Union legislation or*, *where they do not exist*, *upon standards set by Regional Sea Conventions or other international agreements*” [412].

Recognizing that “…*the ecological*, *economic and social consequences of IS [invasive species] in the EU are significant and require a coordinated response*” the EC made in 2008 a formal commitment to develop an EU Strategy on Invasive Alien Species [413]. The EU biodiversity strategy, initially conceived as being achieved by 2020 comprises six targets, one of which relates to invasive alien species (IAS), undertaken to “… *fill policy gaps in combating IAS by developing a dedicated legislative instrument by 2012*” [414]. The legally binding instrument, EU Regulation on the prevention and management of the introduction and spread of invasive alien species, was adopted in 2014 [415]. The regulation imposes restrictions on a list of invasive alien species known as “species of Union concern”. However, the criteria for their selection pose a conundrum: the species shall only be included on the Union list if “*they are … likely to have a significant adverse impact on biodiversity or the related ecosystem services*, *and may also have an adverse impact on human health or the economy*”, and if risk assessment described their “…*adverse impact on biodiversity and related ecosystem services*, *including on native species*, *protected sites*, *endangered habitats*, *as well as on human health*, *safety*, *and the economy including an assessment of the potential future impact having regard to available scientific knowledge*” [415]. Yet, it is compulsory “…*that the inclusion on the Union list will effectively prevent*, *minimise or mitigate their adverse impact*” [415]. Since existing data on marine NIS impacts are scarce, by the time the requisite information is assembled, a given species may have spread and colonized a larger area and thus successful removal, control or containment will likely prove futile [270]. Indeed, only a single estuarine/marine species, the crab *Eriocheir sinensis*, is included in the ‘List of Alien Invasive Species of Union concern’ [144].

### Other regions

In other international, regional-level responses (besides the EU; see Table 8), NIS were considered only marginally without clear demand for actions or appropriate follow-up mechanisms, resulting in a lack of efficacious actions [416–421]. There are several national-level responses, including those in the Canada and US, and Australia and New Zealand [213,422–425], which are largely consistent with those of the IMO (as above) and carry separate enforcement and some cross-border coordination.

**Table 8:** Selected management responses to non-indigenous marine species, by international organizations, in chronological order of response.

Organisation	Established	Management response	Action, reference
International Council for the Exploration of the Sea (ICES)	1902	1969 (Working Group of Non-indigenous Marine Organisms established)	Code of Practice to reduce the risks of adverse effects arising from introduction of non-indigenous marine species [384]
The Convention on Conservation of Nature in the South Pacific	1976 (entry into force 1990)	1976	Apia Convention [416]
United Nations	1982	1982	Convention on the Law of the Sea (UNCLOS) [380]
Barcelona Convention	1976	1982	Protocol Concerning Mediterranean Specially Protected Areas and Biological Diversity in the Mediterranean [417]
Nairobi Convention	1985 (entry into force 1996)	1985	Protocol Concerning Protected Areas and Wild Fauna and Flora in the Eastern African Region [418]
Caribbean Regional Coordinating Unit	1981	1990 (entry into force 2000)	Protocol Concerning Protected Areas and Wildlife in the wider Caribbean Region [419]
The Antarctic Treaty	1959 (entry into force 1961)	1991 (entry into force 1998)	Protocol on Environmental Protection to the Antarctic Treaty, [420]
International Maritime Organisation (IMO)	1948	1991	Guidelines for preventing the introduction of unwanted aquatic organisms and pathogens from ships' ballast water and sediment discharges [390]
Convention on Biological Diversity (CBD)	1992	1992	[381]
IMO	1948	2004 (entry into force 2017, but see text)	Convention for the Control and Management of Ships’ Ballast Water and Sediments [393]
United Nations Environmental Programme (UNEP)	1972	2005	Action Plan concerning species introductions and invasive species in the Mediterranean Sea [426]
International Union for Conservation of Nature (IUCN)	1948	2006	Alien Species in Aquaculture: Considerations for responsible use [389]
Baltic Sea Environmental Protection Commission (HELCOM)	1974	2007	Baltic Sea Action Plan [427]
European Commission (EC)	1992	2007	Regulation concerning use of alien and locally absent species in aquaculture [403]
EC	1992	2008	Marine Strategy Framework Directive [5]
Oslo and Paris Commission (OSPAR)	1972	2008	The General Guidance on the Voluntary Interim application of the D1 Ballast Water Exchange Standard [428]
UNEP	1972	2008	New strategic direction for the Coordinating Body on the Seas of East Asia COBSEA (2008–2012) [420]
IUCN	1948	2009 (Invasive Species Specialist Group established 1993)	Marine Menace—Alien invasive species in the marine environment [429]
IMO	1948	2011	Guidelines for the control and management of ships' biofouling to minimize the transfer of invasive aquatic species [395]
EU	1992	2014	Regulation on invasive species [414]
Arctic Council	1996	2017	Arctic invasive alien species strategy and action plan [430]

## The future is now

**Fig 4:**
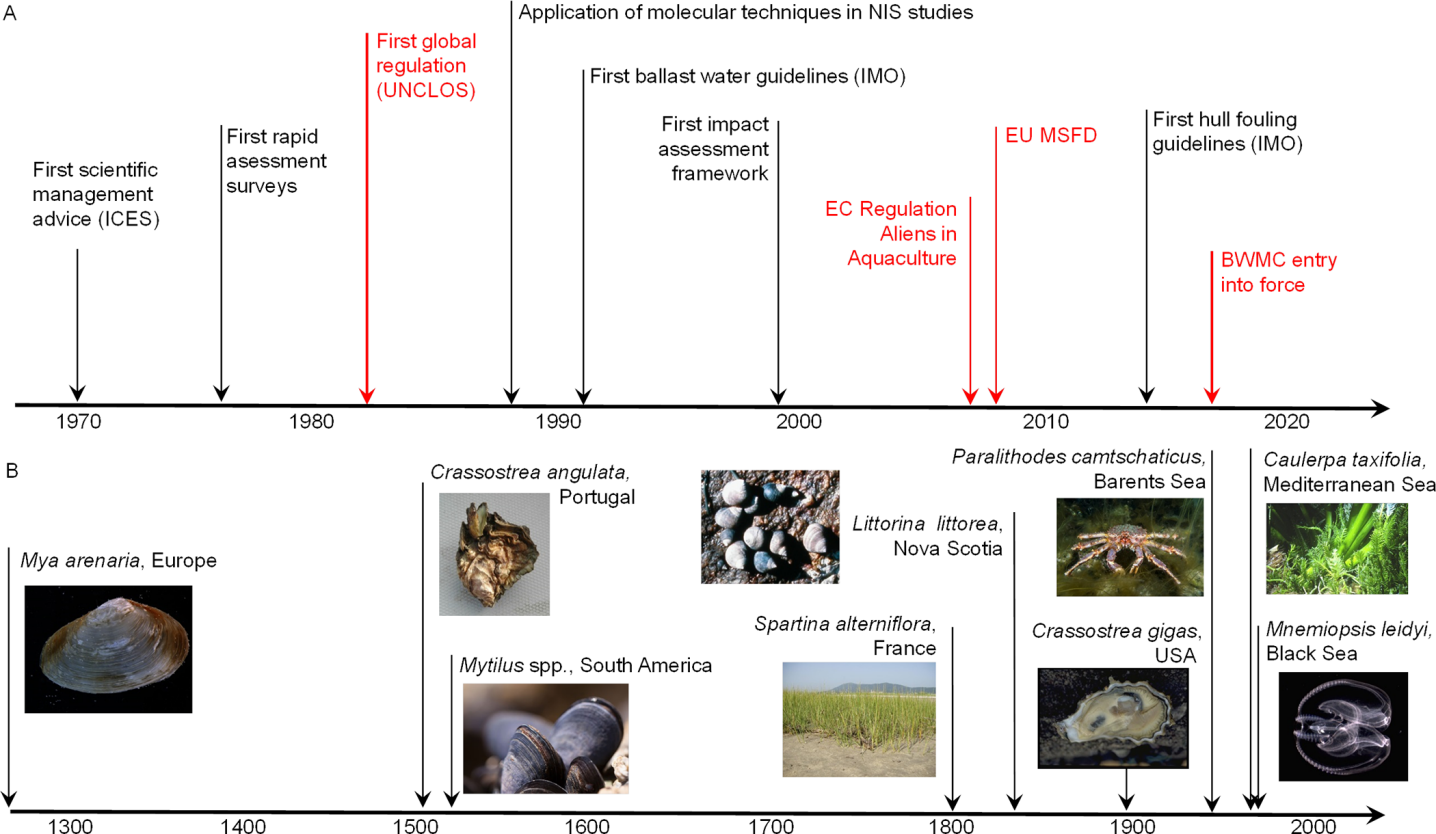
Milestones of management responses to marine bioinvasions, in red–legally binding instruments (panel A); key non-indigenous species introductions since 1200s (panel B). For details, see Global policy and legislation and Table 8. Photo credits: Jim Carlton, IFREMER (France), IMR (Norway), Lauri Laitila, Maiju Lehtiniemi and Pixabay.

Human perception of marine NIS introductions changed in the mid-20^th^ century, following several conspicuous outbreaks that resulted in negative environmental, economic, and public health outcomes. Since then, the role of NIS in biodiversity, habitat and ecological community erosion and the loss of ecosystem services, together with increasing management costs, has been widely recognized. Historical milestones reveal that: i) some marine bioinvasions are millennia-old, ii) the drivers of marine introductions have greatly intensified, diversified and accelerated in recent decades, iii) NIS community baselines vary by region, taxa and time scale, iv) regulatory policies and instruments have been reactive and slow to evolve, attempting to address only a subset of vectors and factors that drive invasions, and v) most major introduction pathways lack legally binding, timely implemented, and strictly monitored instruments (see also Fig 4). Not surprisingly, therefore, the milestones of "2020" noted above for robust NIS action and management cannot now be realized.

It is our sincere conviction that protecting marine ecosystems from further disturbance and preventing socio-economic damages requires urgent changes and advances in the response to NIS, from global to local scales, to minimize invasion impacts. We call for the following key actions:

1. Recognize and acknowledge that effective marine ecosystem management must address both NIS introductions and their interactions with other human stressors (e.g., pollution, fisheries, physical degradation), given that the latter affect invasion dynamics and impacts.
2. Adopt management strategies at multiple spatial scales (as below) that consider the shifting global landscape of invasion risks, due to changing climate and human responses (e.g., changing trade routes/volumes and coastal infrastructure), affecting patterns of propagule delivery, likelihood of invasions, and consequences.
3. Create integrative and comprehensive legal instruments that control the transfer of species by the diverse range of existing and possible future vectors, in order to move beyond the current single vector approach that ignores the multi-vectic nature of both primary and secondary introductions.
4. Provide a robust legal base to enforce controls on species transfers by vectors at both international and regional or national levels. We suggest that the regional sea / large marine ecosystem (or similar) management bodies would be especially instrumental in the implementation of international obligations/legislative acts and the coordination/harmonisation of countries’ responsibilities.
5. Assess the performance of existing and new NIS legal instruments by documenting the rate of new introductions, secondary spread of established NIS populations, and the implementation (management and enforcement). Such performance measures should be a required component of legal instruments, to evaluate efficacy and whether modification (i.e., adaptive management) is needed to meet management objectives.

Without these critical steps to address conspicuous and existing gaps, invasions will remain a major force of change in coastal marine ecosystems, impacting many dimensions of ecosystem function and human society.
